# Editorial: Innovative molecular therapeutic approaches in urothelial carcinoma

**DOI:** 10.3389/fonc.2024.1430285

**Published:** 2024-05-21

**Authors:** Sheldon L. Holder, Ming Yin, Monika Joshi

**Affiliations:** ^1^ Legorreta Cancer Center, Department of Pathology and Laboratory Medicine, Brown University, Providence, RI, United States; ^2^ Comprehensive Cancer Center, Ohio State University, Columbus, OH, United States; ^3^ Penn State Cancer Institute, Division of Hematology/Oncology, Hershey, PA, United States

**Keywords:** urothelial cell carcinoma (UCC), nectin 4, enfortumab vedotin, fibroblast growth factor receptor (FGFR), erdafitinib, HER2, Bacille Calmette-Guérin (BCG), antibody drug conjugate (ADC)

Urothelial cell carcinoma (UCC) is a common cancer, with over 80,000 estimated new cases in the United States of America (USA) in 2024 (1). There continues to be a greater than 3:1 preference for men, with bladder cancer being 4^th^ in incidence in men and representing over 60,000 cases. In contrast, the incidence of bladder cancer in women is estimated to be approximately 20,000 new cases in the USA, which will not place it in the top 10 cancers in women (1).

The median age of diagnosis of UCC is 73 (2), thus, it is common for UCC patients to present with co-morbidities that influence treatment choices. Use of chemotherapy is common for muscle invasive disease and is also used for non-muscle invasive disease. Increasingly, personalized medicine is driving treatment choices away from non-specific chemotherapy and towards targeted therapies in an effort to increase efficacy and decrease adverse effects. In this Research Topic we assemble articles that discuss therapeutic targets in UCC. The Research Topic consists of three review articles that discuss antibody drug conjugates (ADCs) and biomarkers in urothelial carcinoma, and three research articles that span non-muscle invasive, muscle-invasive, and upper tract UCC.

In the review, *Antibody-drug conjugates and predictive biomarkers in advanced urothelial carcinoma*, Fenton and VanderWeele discuss several ADCs that are currently being studied in UCC. The review includes an informative and thorough list of ongoing ADC trials in UCC. Furthermore, the authors discuss potential biomarkers for targeted therapy in UCC, including nectin-4, TP53, CDKN2B, Trop-2, Topoisomeraise-1, and HER2.

Of the ADCs being evaluated in UCC, enfortumab-vedotin (EV) is the most used. In *Enfortumab vedotin in metastatic urothelial carcinioma: the solution Eventually*?, Maiorano et al. provide an in-depth examination of the activity of EV in metastatic UCC. In the review, readers will learn the pharmacology, pertinent completed clinical trials, placement of treatment with EV relative to other approved therapies, safety signals, and management of adverse effects. A very useful table of current, open EV studies is also included. This review of EV in metastatic UC is particularly timely given the recent data showing EV in combination with pembrolizumab to be superior to platinum-based chemotherapy in patients with metastatic UCC (3).

While targeting HER2 in UCC is not new, to date it has met with mixed results. In *HER2 expression in urothelial carcinoma, a systemic literature review*, Scherrer et al. seek to determine the prevalence of HER2 expression in UCC from the published body of literature. The analysis includes a modern classification: HER2 positive, HER2 low, and HER2 negative. Additional important considerations assessed are the method of HER2 evaluation (expression, gene amplification, or mutation), and concordance of different testing methods.

The Research Topic considers narrower scopes of investigation within different types of UCC. Intravesical instillation of Bacillus Calmette-Guerin (BCG) is commonly used to treat non-muscle invasive UCC. In the original article, *Antibiotic therapy impact on intravesical BCG therapy efficacy for high-risk localized bladder cancer treatment*, Aubert et al. perform a retrospective analysis to determine the degree to which antibiotic use affects the efficacy of BCG. Consideration is given to duration or delay of treatment, and effects on recurrence and progression.

Upper tact UCC is often treated similarly to bladder UCC, but the development of targeted therapies in upper tract UCC is even more scarce than in bladder UCC. In *Clinicopathological characteristics and loss of mismatch repair protein expression in Chinese upper tract urothelial carcinomas*, Shang et al. explore the implications of loss of DNA mismatch repair functionality in a Chinese cohort of patients. Rates of mismatch repair (MMR) loss, and the effects on T-stage and survival are examined. Comparison is also made to a Western cohort.

The Research Topic is completed with an assessment of fibroblast growth factor receptor (FGFR) targeted therapy in metastatic UCC. The FGFR inhibitor, erdafitinib, is active in FGFR altered metastatic UCC (4). In *Erdafitinib treatment in metastatic urothelial carcinoma: a real-world analysis*, Rouvinov et al. report the clinical benefit and safety profile of erdafitinib in a real-world setting.

In the Research Topic, *Innovative molecular therapeutic approaches in urothelial carcinoma*, we provide pertinent articles examining ADCs, HER2, FGFR, MMR, and the effect of antibiotic use in UCC. As laboratory researchers and clinical investigators increase focus on molecularly targeted therapies, we will find more efficacious and tolerable therapies for UCC.

## Author contributions

SH: Writing – review & editing, Writing – original draft. MY: Writing – review & editing. MJ: Writing – review & editing.

## References

[1] Siegel RL Giaquinto AN Jemal A. Cancer Statistics. CA Cancer J Clin. (2024) 74:12–49. doi: 10.3322/caac.21820 38230766

[2] Cancer stat facts: bladder cancer, in: NIH NCI: surveillance, epidemiology, and end results program . Available online at: https://seer.cancer.gov/statfacts/html/urinb.html (Accessed 2024 April 22).

[3] Powles T Valderrama BP Gupta S Bedke J Kikuchi E Hoffman-Censits J. Enfortumab vedotin and pembrolizumab in untreated advanced urothelial cancer. N Engl J Med. (2024) 390:875–88. doi: 10.1056/NEJMoa2312117 38446675

[4] Siefker-Radtke AO Necchi A Park SH Garcia-Donas J Huddart RA Burgess EF. Efficacy and safety of erdafitinib in patients with locally advanced or metastatic urothelial carcinoma: long-term follow-up of a phase 2 study. Lancet Oncol. (2022) 23:248–58. doi: 10.1016/S1470-2045(21)00660-4 35030333

